# Zika virus infection preceding Guillain–Barré syndrome: Two cases from Southern India

**DOI:** 10.1177/00494755261437243

**Published:** 2026-03-29

**Authors:** Amrita Pattanaik, Nidhi Ashtaputre, Reeta S Mani, Chiranjay Mukhopadhyay, Aparna R Pai

**Affiliations:** 1 Assistant Professor, Department of Virus Research, Manipal Institute of Virology, Manipal Academy of Higher Education 444442 , Manipal, Karnataka, India; 2 Student, Department of Virus Research, 29148 Manipal Institute of Virology, Manipal Academy of Higher Education, Manipal, Karnataka, India; 3 Professor, Department of Neurovirology, 29224 National Institute of Mental Health and Neurosciences, Bengaluru, Karnataka, India; 4 Professor, Department of Virus Research, Manipal Institute of Virology, Manipal Academy of Higher Education, Manipal, Karnataka, India; 5 Professor, Department of Neurology, Kasturba Medical College, Manipal Academy of Higher Education, Manipal, Karnataka, India

**Keywords:** Encephalitis < disease, Asia < location, public health, diagnosis < other, disease prevention < disease

## Abstract

Guillain–Barré syndrome (GBS) is a leading cause of acute flaccid paralysis and has been associated with several arboviral infections. It is an acute immune-mediated polyradiculo-neuropathy characterised by rapidly progressive limb weakness, areflexia, and variable cranial nerve involvement. Antecedent infections are reported in the majority of patients, most commonly gastro-intestinal or respiratory. In recent years, arboviruses such as Dengue, Chikungunya, and Zika have emerged as important infectious triggers of GBS, particularly in tropical regions. Zika virus infection is often asymptomatic or presents with non-specific febrile illness, making it likely to be under-diagnosed in endemic settings.

## Case report 1

The first patient was a 54-year old man who presented with acute onset ascending weakness involving all four limbs, accompanied by dysphagia and respiratory difficulty.^1,2^ He reported a self-limited febrile illness eight days prior to the onset of neurological symptoms.^1,2,3^

On examination, he was conscious, oriented, obeying commands, with a Glasgow Coma Scale (GCS) score of 15/15.^4^ Vital signs were stable (pulse 86/min, blood pressure 130/90 mmHg, respiratory rate 16/min, oxygen saturation 99% on room air) Neurological assessment revealed quadriparesis with depressed deep tendon reflexes and no initial cranial nerve involvement.^5^ Cerebrospinal fluid (CSF) analysis demonstrated albumino-cytological dissociation.^2^ Nerve conduction studies were consistent with acute inflammatory demyelinating polyradiculo-neuropathy.

During his admission, there was rapid neurological deterioration with bulbar and respiratory failure, necessitating mechanical ventilation and tracheostomy.

Intravenous immunoglobulin therapy was given with gradual neurological improvement. Serum testing revealed IgM antibodies against Zika virus, while testing for other common arboviral and viral triggers of GBS was negative. At discharge, the patient was able to stand with support and was referred for rehabilitation.

## Case report 2

The second patient was a 52-year old woman who presented with a six-day history of progressive limb weakness and dysphagia. On examination she was conscious, oriented, obeying commands, with a Glasgow Coma Scale (GCS) score of 15/15. She was haemodynamically stable. Her peripheral capillary oxygen saturation (SpO2) was 93% on room air. Neurological assessment showed quadriparesis with bilateral lower moto neurone facial palsy and palatal weakness. CSF analysis demonstrated albumino-cytological dissociation.^2^ Initial nerve conduction studies suggested an evolving neuropathy, and repeat studies fulfilled demyelinating criteria consistent with acute inflammatory demyelinating polyradiculo-neuropathy.

She also developed respiratory compromise and needed high-dependency unit care. Intravenous immunoglobulin therapy was administered, following which she showed marked clinical improvement. Serological testing detected IgM antibodies against Zika virus, while testing for other viral causes of GBS was negative. At discharge, she was ambulant with mild residual weakness. A comparative summary of the clinical features of both patients is presented in Table 1.

**Table 1.** Summary of clinical features of two patients with Guillain–Barré syndrome and Zika virus infection.

Parameter	Case 1	Case 2
Age / Sex	54 years / Male	52 years / Female
Antecedent illness	Febrile illness with cough	None reported
Neurological features	Quadriparesis, later bulbar and respiratory weakness	Quadriparesis, facial palsy, bulbar weakness
CSF findings	Elevated protein, normal cell count	Elevated protein, normal cell count
Nerve conduction studies	Demyelinating pattern (AIDP)	Demyelinating pattern (AIDP)
Zika virus serology	Anti-Zika IgM positive	Anti-Zika IgM positive
Treatment	IVIG, ICU care, mechanical ventilation	IVIG, high-dependency care
Outcome at discharge	Able to stand with support	Independent ambulation with mild weakness

## Discussion

Serum samples from both patients were tested for Zika virus RNA by reverse transcription PCR, which was negative. Anti-Zika IgM antibodies were detected using a commercially available IgM capture ELISA (ZIKV Detect™ 2.0 IgM Capture ELISA; InBios International, Seattle, USA). The assay incorporates cross-reactive control antigen and normal cell antigen to help distinguish Zika virus infection from other flaviviruses. Both samples fulfilled the manufacturer's criteria for presumptive Zika positivity based on Zika antigen optical density and immune status ratio. To address possible flavivirus cross-reactivity, serum samples were also tested for IgM antibodies against other arboviruses endemic to the region, including dengue, chikungunya, Japanese encephalitis and West Nile using commercially available ELISA assays. These tests were negative in both patients. We did not, however, test for bacterial pathogens such as *Campylobacter jejuni*, a recognised antecedent trigger of Guillain–Barré syndrome. This represents a limitation of the study.

Zika virus–associated GBS has been well documented during outbreaks in South America and the Pacific, most notably in French Polynesia, where a strong temporal association was demonstrated.^6^ Subsequent systematic reviews and meta-analyses have confirmed an increased risk of GBS following Zika virus infection.^7^ Proposed mechanisms include immune-mediated nerve injury driven by molecular mimicry between viral antigens and peripheral nerve components.^2^ In India, sporadic Zika outbreaks have been reported, but systematic testing for Zika virus is not routinely performed in patients with GBS.^8,9^ No confirmed Zika outbreak was reported in the region during the study period. However, sporadic arboviral infections of Dengue, Chikungunya and Japanese encephalitis are endemic.

Both patients described developed severe neurological disease requiring advanced supportive care, consistent with reports indicating more severe clinical courses in Zika-associated GBS compared with non-Zika-related cases.^10^ Given the co-circulation of multiple arboviruses and the potential for serological cross-reactivity, the use of diagnostic assays capable of differentiating Zika virus antibodies is essential.^5^ Although Zika virus RNA was not detected by RT-PCR, both patients presented during the neurological phase of illness several days after the antecedent infection. Zika viraemia is typically transient and may no longer be detectable at this stage, making IgM serology the more informative diagnostic test. These cases highlight the importance of considering Zika virus infection in the diagnostic evaluation of GBS in tropical settings.
